# Using advanced analysis of multifocal visual-evoked potentials to evaluate the risk of clinical progression in patients with radiologically isolated syndrome

**DOI:** 10.1038/s41598-021-81826-z

**Published:** 2021-01-21

**Authors:** J. M. Miguel, M. Roldán, C. Pérez-Rico, M. Ortiz, L. Boquete, R. Blanco

**Affiliations:** 1 Biomedical Engineering Group, Department of Electronics, University of Alcalá, 28805 Alcalá de Henares, Madrid, Spain; 2 Department of Ophthalmology, Príncipe de Asturias University Hospital, Madrid, Spain; 3 Department of Surgery, Medical and Social Sciences, University of Alcalá, Carretera Alcalá-Meco S/N, 28805 Alcalá de Henares, Madrid, Spain; 4 School of Physics, University of Melbourne, Melbourne, VIC 3010 Australia; 5 Ramón Y Cajal Health Research Institute (IRYCIS), 28034 Madrid, Spain

**Keywords:** Neuroscience, Multiple sclerosis

## Abstract

This study aimed to assess the role of multifocal visual-evoked potentials (mfVEPs) as a guiding factor for clinical conversion of radiologically isolated syndrome (RIS). We longitudinally followed a cohort of 15 patients diagnosed with RIS. All subjects underwent thorough ophthalmological, neurological and imaging examinations. The mfVEP signals were analysed to obtain features in the time domain (SNR_min_: amplitude, Lat_max_: monocular latency) and in the continuous wavelet transform (CWT) domain (b_max_: instant in which the CWT function maximum appears, N_max_: number of CWT function maximums). The best features were used as inputs to a RUSBoost boosting-based sampling algorithm to improve the mfVEP diagnostic performance. Five of the 15 patients developed an objective clinical symptom consistent with an inflammatory demyelinating central nervous system syndrome during follow-up (mean time: 13.40 months). The (**SNR**_**min**_) variable decreased significantly in the group that converted (2.74 ± 0.92 vs. 4.07 ± 0.95, *p* = 0.01). Similarly, the **(b**_**max**_) feature increased significantly in RIS patients who converted (169.44 ± 24.81 vs. 139.03 ± 11.95 (ms), *p* = 0.02). The area under the curve analysis produced **SNR**_**min**_ and **b**_**max**_ values of 0.92 and 0.88, respectively. These results provide a set of new mfVEP features that can be potentially useful for predicting prognosis in RIS patients.

## Introduction

The afferent visual system is affected very frequently and at a very early stage in demyelinating processes. Consequently, study of it may lead to broader and deeper understanding of these neurological pathologies^1,2^. Diagnosis and evaluation over time of the largely subclinical nature of defects of the afferent visual pathway in demyelinating processes require the use of innovative structural and functional diagnostic technologies such as latest-generation optical coherence tomography (OCT) and multifocal visual-evoked potentials (mfVEPs), respectively^3^.

The increasing use of magnetic resonance imaging (MRI) to evaluate clinical pictures such as migraine, dizziness or vertigo has led to the emergence of a new clinical entity—radiologically isolated syndrome (RIS)—within demyelinating processes’ clinical spectrum^4^. This syndrome is characterized by the detection in MRI of lesions in the white matter of the central nervous system (CNS) that, due to their size, location and morphology, are highly suggestive of demyelinating plaques exhibiting dissemination in space (DIS) in subjects presenting with normal neurological examination results and no history of signs or symptoms of multiple sclerosis (MS)^4^. The recently published MAGNIMS consensus recommendations^5^ propose using the same DIS criterion, as set out in the latest review of the McDonald criteria^6^, to diagnose RIS and MS.

Since RIS was first described there has been great interest in establishing the risk of the syndrome evolving into MS in these subjects. According to various studies, some patients diagnosed with RIS will eventually progress to MS, suggesting that this syndrome may represent a preclinical stage of MS, at least in some cases^7,8^. Recent studies suggest that patients with RIS, clinically isolated syndrome (CIS) and relapsing–remitting multiple sclerosis (RRMS) all share non-motor clinical characteristics^9,10^ and suffer quantitative brain tissue damage^11^, indicating that RIS evinces MS in its early, preclinical form.

The clinical evolution of patients who meet the diagnostic criteria for RIS is uncertain^12^. It is therefore important to differentiate between those subjects at high risk of suffering demyelinating clinical events and being diagnosed with CIS, RRMS or primary progressive multiple sclerosis (PPMS) and those with static lesions or lesions due to other aetiologies. A subject with RIS can remain asymptomatic and present a stable MRI, develop new lesions in follow-up MRIs while remaining asymptomatic, or present a first clinical event typical of MS, i.e. CIS or even PPMS or RRMS.

Clinical management of RIS patients, who meet DIS criteria in MRI scans but show no symptoms, remains a major challenge in clinical practice as there is a lack of scientific evidence relating to this pathology^13,14^.

To the best of our knowledge, no studies have been conducted into the role of mfVEPs in RIS patients and evaluation of the risk of conversion of RIS to CIS/MS. MfVEPs objectively evaluate visual function and the integrity of the optical pathway^15^ and have been used to study various optic nerve and ganglion cell diseases^16^. In this technique, the visual stimulus is usually subdivided into a number of sectors (typically 60). Each of these sectors is independently stimulated using specialized software. The electrical activity evoked in the visual cortex by each stimulus is recorded in electroencephalograms (EEGs). From a single, continuous EEG signal, a mathematical algorithm extracts the evoked response generated by each sector^17,18^.

Traditional analysis of mfVEP recordings is based on the study of the recordings’ amplitudes and latencies^16,18^. However, it has been demonstrated that in some cases diagnosis using mfVEP signals can be improved using advanced signal filtering and extraction algorithms, such as the wavelet transform^19^, empirical mode decomposition^20^, and singular spectrum analysis^21^, among other alternatives.

The goal of our study was to assess the role of multifocal visual-evoked potentials as a guiding factor for RIS subject conversion to CIS/MS.

## Material and methods

Fifteen asymptomatic subjects (13 females, 2 males; mean age 38.9 years; range 19.7–50.0 years) were enrolled in the study. All fulfilled the recently identified criteria for RIS which imply that none of the subjects had previously experienced remitting clinical symptoms consistent with neurological dysfunction of the CNS^12^. All subjects were consecutively contacted by the same neurologist. At baseline, we recorded their detailed historical and current clinical data and key episodes in the course of their RIS. Comprehensive neurological and ophthalmological examinations and structural neuro-imaging of the brain and spinal cord were performed.

All subjects were examined using an identical MRI protocol. Brain MRI scans were obtained in a single session using a Philips Gyroscan operating at 1.5 T (Philips Medical Systems, Best, The Netherlands). All participants presented asymptomatic T2-hyperintense brain lesions greater than 3 mm in diameter fulfilling the Barkhof criteria^22^. None of them had experienced neurological symptoms suggestive of clinical manifestation of MS.

The study protocol was approved by the University Hospital Principe de Asturias Review Board and adhered to the tenets of the Declaration of Helsinki, and all participants provided their informed consent. Data were kept in accordance with Spanish Law 15/1999 on data protection to protect patient confidentially.

### Multifocal visual-evoked potential recordings

As previously described^20,23^, mfVEP signals were recorded monocularly with VERIS software 5.9 (Electro-Diagnostic Imaging, Inc., Redwood City, CA). The visual stimulus was a scaled dartboard with a diameter of 44.5 degrees, containing 60 sectors, each with 16 alternating checks. The luminance for the white and black checks was 200 and < 3 cd/m^2^, respectively. The checks in each sector were reversed in contrast using a pseudorandom sequence at a frame rate of 75 Hz. The signals were amplified at a gain of 10^5^ at a bandwidth between 3 and 100 Hz. The sampling frequency was 1200 Hz, obtaining 600 samples in each recording (length 500 ms). The signals were digital-passband-filtered (1–35 Hz) using the fast Fourier transform. Three channels were obtained for each sector from the differences between the active electrodes and the reference electrode, along with three additional derived channels. Each channel was divided into two different intervals: the signal window (45–150 ms), which contains the evoked potential response, and the noise window (325–430 ms), which essentially contains noise^21^.

### Multifocal VEP response analysis and classification

Analysis of the mfVEP recordings was performed in the time domain (amplitude and latency) and the continuous wavelet transform (CWT) domain (variables b_max_ and N_max_).

The amplitude of the mfVEP recording was quantified as the signal-to-noise ratio (SNR), calculated as^18^:

1 $${\text{SNR}}\left( {{\raise0.7ex\hbox{${\text{V}}$} \!\mathord{\left/ {\vphantom {{\text{V}} {\text{V}}}}\right.\kern-\nulldelimiterspace} \!\lower0.7ex\hbox{${\text{V}}$}}} \right) = \frac{{{\text{RMS}}\left( {{\text{X}}_{{45 - 150{\text{ ms}}}} } \right)}}{{{\text{mean}}\left( {{\text{RMS }}\left( {{\text{X}}_{{325 - 430{\text{ ms}}}} } \right)} \right)}}$$

where RMS(X_45–150 ms_) was the root mean square (RMS) amplitude of the waveform in the signal window. The mean RMS(X_325–430 ms_) was the average RMS amplitude of all 60 waveforms in the noise windows**.** In each of the sectors, only the best channel (i.e. the one with the highest SNR) was analysed^21^.

For each study subject eye the following time domain features were obtained from the mfVEP signals: (1) **SNR** (dimensionless: V/V)**:** which corresponds to the mean value in all sectors of an eye with the amplitude of the signals (Eq. 1) and; (2) mean **monocular latency value (ms)** of all sectors of each eye. In each sector, monocular latency was obtained by finding the instant of maximum correlation with the normative database^17^.

The CWT of a time signal, x(t), is defined as^24^:

2 $${\text{T}}\left( {{\text{a}},{\text{b}}} \right) = \frac{1}{{\sqrt {\text{a}} }}\mathop \smallint \limits_{ - \infty }^{ + \infty } {\text{x}}\left( {\text{t}} \right)\Psi ^{*} \left( {\frac{{{\text{t}} - {\text{b}}}}{{\text{a}}}} \right){\text{dt}}$$

where Ψ*(t) is the complex conjugate of wavelet function Ψ(t) (real Daubechies 7 wavelet: db7); a (dimensionless) is the dilation parameter of the wavelet; and b (ms) is the translation parameter (a,b ∈ R; a ≠ 0). As the CWT can describe time and frequency components of a signal in detail, it is possible to obtain new mfVEP signal descriptors that could constitute electrophysiological biomarkers.

For each sector, the best channel was selected and the CWT modulus was obtained: |T(a,b)|. The following features were calculated: Translation **b**_**max**_ (ms) at which the absolute maximum value (max|T(a,b)|) appears and Number of local maxima (**N**_**max**_) (dimensionless) in |T(a,b)| that exceed (max|T(a,b)|)/3. The mean value of these variables was then obtained in the 60 sectors of each eye.

For each patient, the following variables extracted from the time analysis and CWT were considered: (1) **SNR**_**min**_**:** SNR value of that patient's eye with least amplitude; (2) **Lat**_**max**_: monocular latency of the eye that presents the greatest delay in the evoked response; (3) Translation (**b**_**max**_) of the eye that presents the highest value in the wavelet domain; (4) Number of local maxima (**N**_**max**_) of the eye that presents the highest value in the wavelet domain. The variables that best identified the patients who converted clinically were selected.

*RUSBoost*^25^ is a hybrid data sampling/boosting algorithm designed to improve the performance of models trained on skewed data. The boosting process assigns greater weights to misclassified examples, which are usually the minority class examples. RUSBoost is especially effective at classifying imbalanced data, as in our case, in which the relationship between patients who convert (RIS_conv) and those who do not (RIS_non_conv) is 1/3.

### Statistical analysis

Statistical analyses were performed using IBM SPSS Statistics 25 software (SPSS Inc. Chicago, Illinois, USA).Intergroup comparison was performed with the Fisher exact test for categorical variables and with the t-test or Wilcoxon test for quantitative variables. All tests were 2-tailed and *p* < 0.05 was considered statistically significant. Survival analysis was used to assess time-dependent variables using Kaplan–Meier estimates. The area under the receiver operating characteristic curve (AUC) was employed to assess the discrimination capability for each of the features proposed in this study. The classification process was summarized in a confusion matrix with sensitivity, specificity and ROC (receiver operating characteristic) analysis parameters.

## Results

All subjects with RIS presented normal neurological examination results and conventional MRI scans, and the Barkhof criteria were confirmed in all patients. Table 1 summarizes the baseline demographic and the clinical and radiological characteristics of the RIS study cohort, including the reason for the first MRI scan. The study cohort principally comprised women (13/15). Mean age was 38.9 years, range 19.7–50 years. A positive family history of MS was not observed in any member of the study group. All subjects had expanded disability status scale scores of 0.0 on the initial baseline examination. Reasons for the initial MRI brain scan identifying CNS anomalies suggestive of demyelinating disease were migraine (33.3%), vertigo (20%), tinnitus (13.3%), anosmia (6.6%) and paraesthesia (6.6%). None of these complaints were related to a demyelinating event. None of our RIS subjects had undergone any approved disease-modifying therapies before the development of their first clinical event. At baseline, 14 subjects (93.3%) had > 9 T2-hyperintense MRI lesions, 14 (93.3%) had periventricular lesions, 10 (66,6%) had juxtacortical lesions and 1 (6.6%) had infratentorial lesions. Gadolinium-positive lesions and spinal cord lesions were present in 5 (33.3%) and 2 (13.3%) subjects, respectively.

**Table 1:** Summary of RIS subjects’ baseline characteristics (*n* = 15).

Age (mean, SD)	38.9 ± 9.2
Female, n (%)	13 (86.6)
**Medical background**	
Migraine	5 (33.3)
Vertigo	3 (20)
Tinnitus	2 (13.3)
Anosmia	1 (66)
Paraesthesia	1 (66)
**MRI lesions, n (%)**	
≥ 9T2-hyperintense MR imaging lesions, n (%)	14 (93.3)
Periventricular lesions, n (%)	14 (93.3)
Infratentorial lesions, n (%)	1 (6.6)
Juxtacortical lesions, n (%)	10 (66.6)
Spinal cord lesions, n (%)	2 (13.3)
Gd + lesions, n (%)	5 (33.3)

Data in mean ± standard deviation and percentages.

*RIS* radiologically isolated syndrome, *MRI* magnetic resonance imaging.

**Figure 1:**
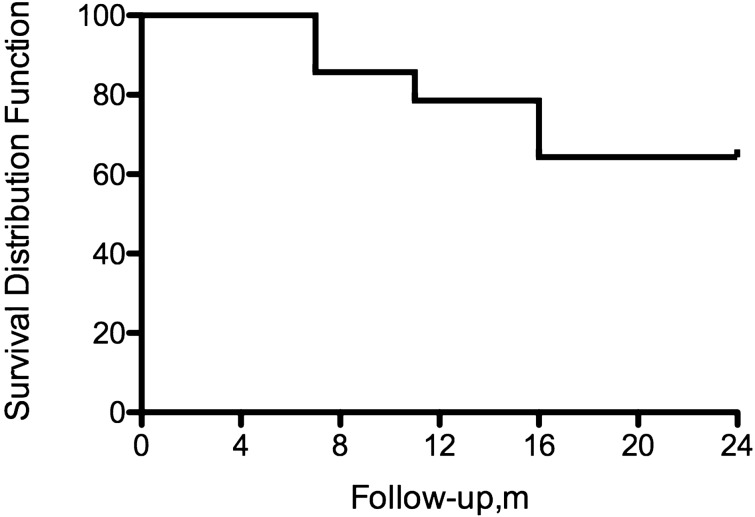
Kaplan–Meier survival curve showing the risk of clinical progression. At 1 year, 73.3% of RIS patients have not experienced CIS/MS conversion. At 2 years, 66.6% of patients have not progressed. This image was generated using Prism (Version: 5, Url: https://www.graphpad.com/).

During the study follow-up period (mean time: 13.40 months; range: 9–19 months), five (5/15; 33.3%) RIS subjects presented radiological and clinical conversion to the following conditions: 3 (60%) presented RRMS; 1 (20%) presented PPMS and 1 (20%) presented CIS syndrome. Survival analysis (Fig. 1) was used to assess time-dependent variables and the endpoint was the time from the first MRI to CIS/MS. The 12-month first acute or progressive clinical event rate in the RIS group was 26.6% (4/15). In all patients experiencing clinical episodes, symptoms proved to be consistent with a demyelinating event.

Subjects who progressed clinically (RIS_conv) (5/15, 33.3%) were significantly younger than those who did not convert (RIS_non_conv): 30.89 ± 7.97 versus 42.96 ± 7.06, *p* = 0.01. No significant differences in high- and low-contrast BCVA (logmar) were found between the eyes of both groups (0.0 ± 0.03 vs. 0.0 ± 0.01; *p* = 0.56). In addition, no significant differences were observed in the total number of white matter lesions in the baseline MRI scan between the two groups (19.2 ± 4.69 vs. 18.2 ± 8.01, *p* = 0.66) or in the number of spinal and gadolinium-enhanced lesions (*p* > 0.25) or other MRI lesions (*p* > 0.28) between the two groups.

**Figure 2:**
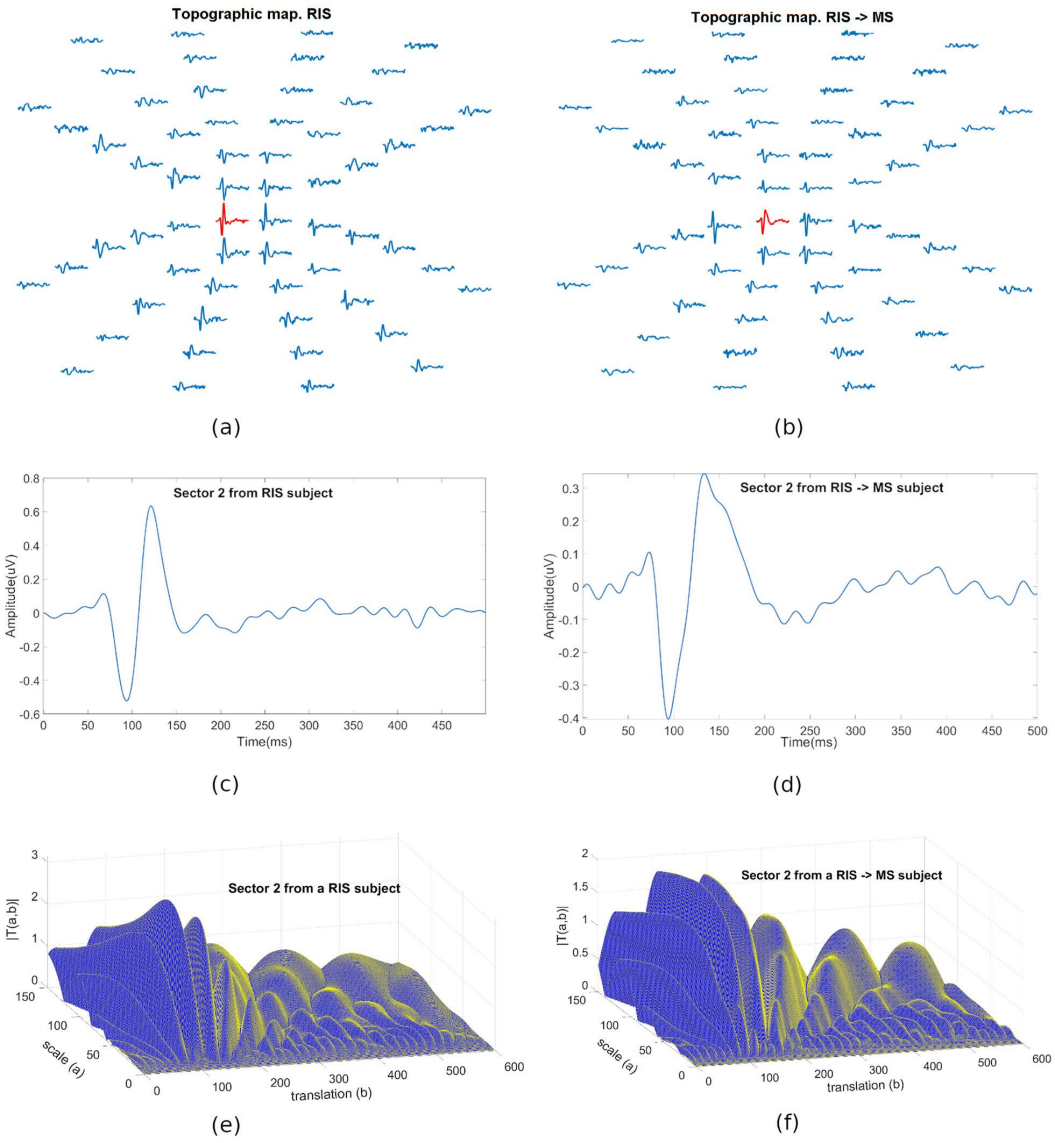
Multi-phase graphical representation of the method implemented. (**a**) mfVEP arrays of an RIS_non_conv patient. (**b**) mfVEP arrays of an RIS_conv patient. (**c**) X(t) signal in the sector marked in red in (**a**). (**d**) X(t) signal in the sector marked in red in (**b**). (**e**) CWT modulus of the signal represented in (**c**). (**f**) CWT modulus of the signal represented in (**d**). These images were generated using Matlab (Version: R2018b, Url: https://www.mathworks.com/products/matlab.html.

Figure 2 shows the mfVEP recordings (array of 60 signals from the best channel) taken from an RIS_non_conv subject (a) and an RIS_conv patient (b). In both cases, it shows in detail an mfVEP signal for a given sector (Fig. 2c,d) and its respective |T(a,b)| functions (Fig. 2e,f). In this particular example, the amplitudes of the RIS_non_conv subject's mfVEP signals are greater than those of the RIS_conv patient.

Table 2 shows the results obtained with the two analysis variables selected in the mfVEP signals’ time domain: SNR_min_, Lat_max_, and the two variables (b_max_ and N_max_) obtained from the CWT analysis to try to identify those RIS subjects at greatest risk of progression. Thus, we observed that the (**SNR**_**min**_) variable decreased significantly among RIS_conv subjects (2.74 ± 0.92 vs. 4.07 ± 0.95, *p* = 0.010). However, for the **Lat**_**max**_ variable (0.22 ± 3.62 vs. 0.55 ± 1.50 ms) we did not observe significant differences between the two groups (*p* = 0.62). At the same time, the (**b**_**max**_) variable increased significantly in the RIS_conv group (169.44 ± 24.81 vs. 139.03 ± 11.95 (ms), *p* = 0.02), and we did not observe significant differences in the **N**_**max**_ variable between the two groups (35.05 ± 15.19 vs. 24.80 ± 9.93; *p* = 0.086).

**Table 2:** Comparison of the variables obtained between the two groups.

mfVEP signal features	RIS_non_conv	RIS_conv	*p* value AUC
SNR_min_ (dimensionless)	4.07 ± 0.95	2.74 ± 0.92	*p* = 0.010 (t‐test) AUC = 0.92
**Lat**_**max**_ (ms)	0.55 ± 1.50	0.22 ± 3.62	*p* = 0.62 (t‐test) AUC = 0.58
**B**_**max**_ (ms)	139.03 ± 11.95	169.44 ± 24.81	*p* = 0.020 (t-test) AUC = 0.88
**N**_**max**_ (dimensionless)	24.80 ± 9.93	35.05 ± 15.19	*p* = 0.086 (W-test) AUC = 0.78

**Figure 3:**
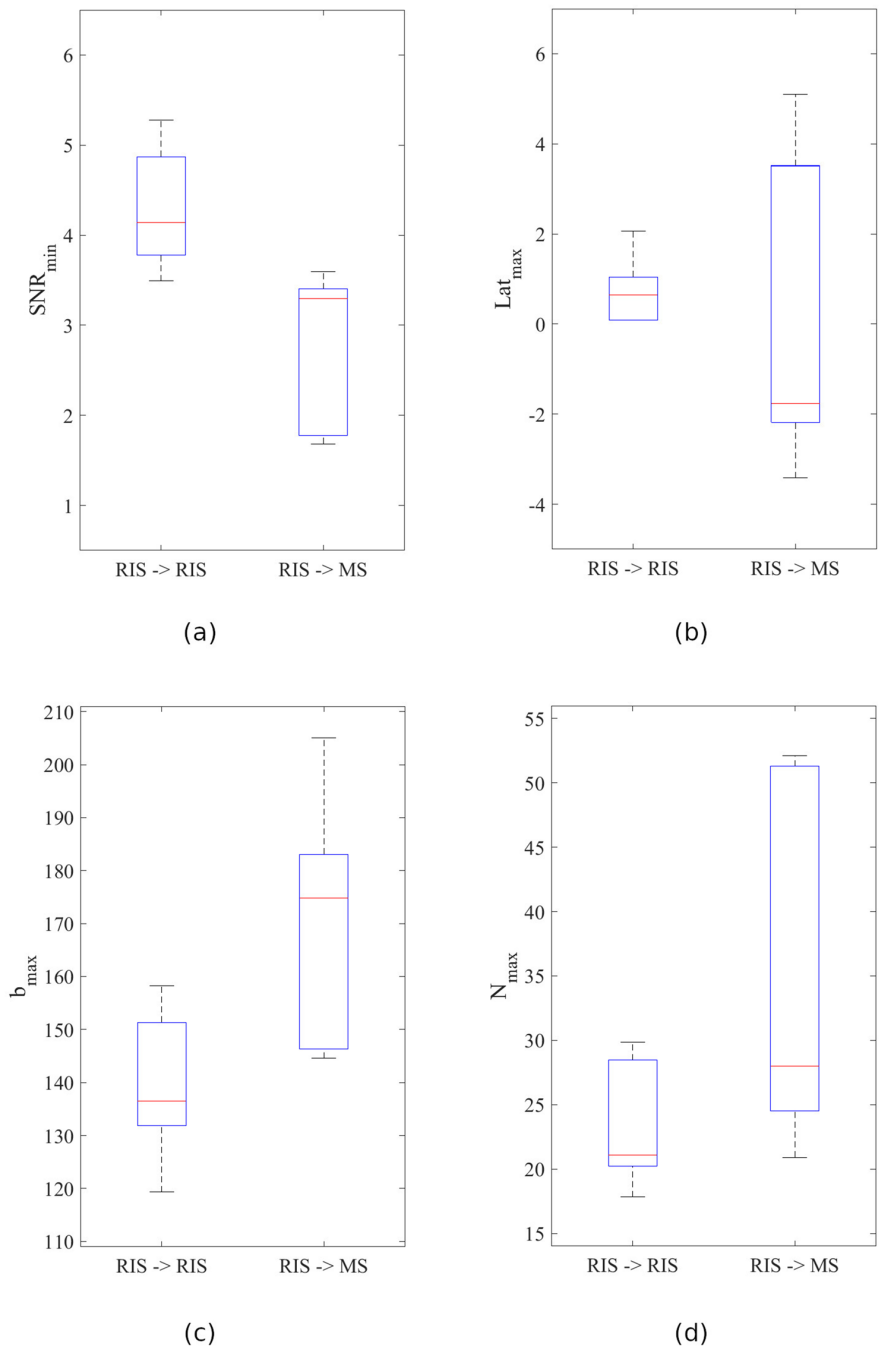
Boxplot of variables included in the study: (**a**) SNR_min_ variable, (**b**) Lat_max_ variable, (**c**) b_max_ variable, (**d**) N_max_ variable. These images were generated using Matlab (Version: R2018b, Url: https://www.mathworks.com/products/matlab.html.

We then evaluated the diagnostic accuracy of these mfVEP signal variables using ROC curve analysis. Thus, the **SNR**_**min**_ and **b**_**max**_ variables obtained AUC values of 0.92 and 0.88, respectively, while the **Lat**_**max**_ and **N**_**max**_ values were lower (0.58 and 0.78, respectively). On average, the two variables obtained in the CWT domain together provide greater diagnostic accuracy (AUC_MEAN_ = 0.83) than the two standard amplitude and latency variables (AUC_MEAN_ = 0.75). Figure 3 shows the boxplots of the four features studied.

**Figure 4:**
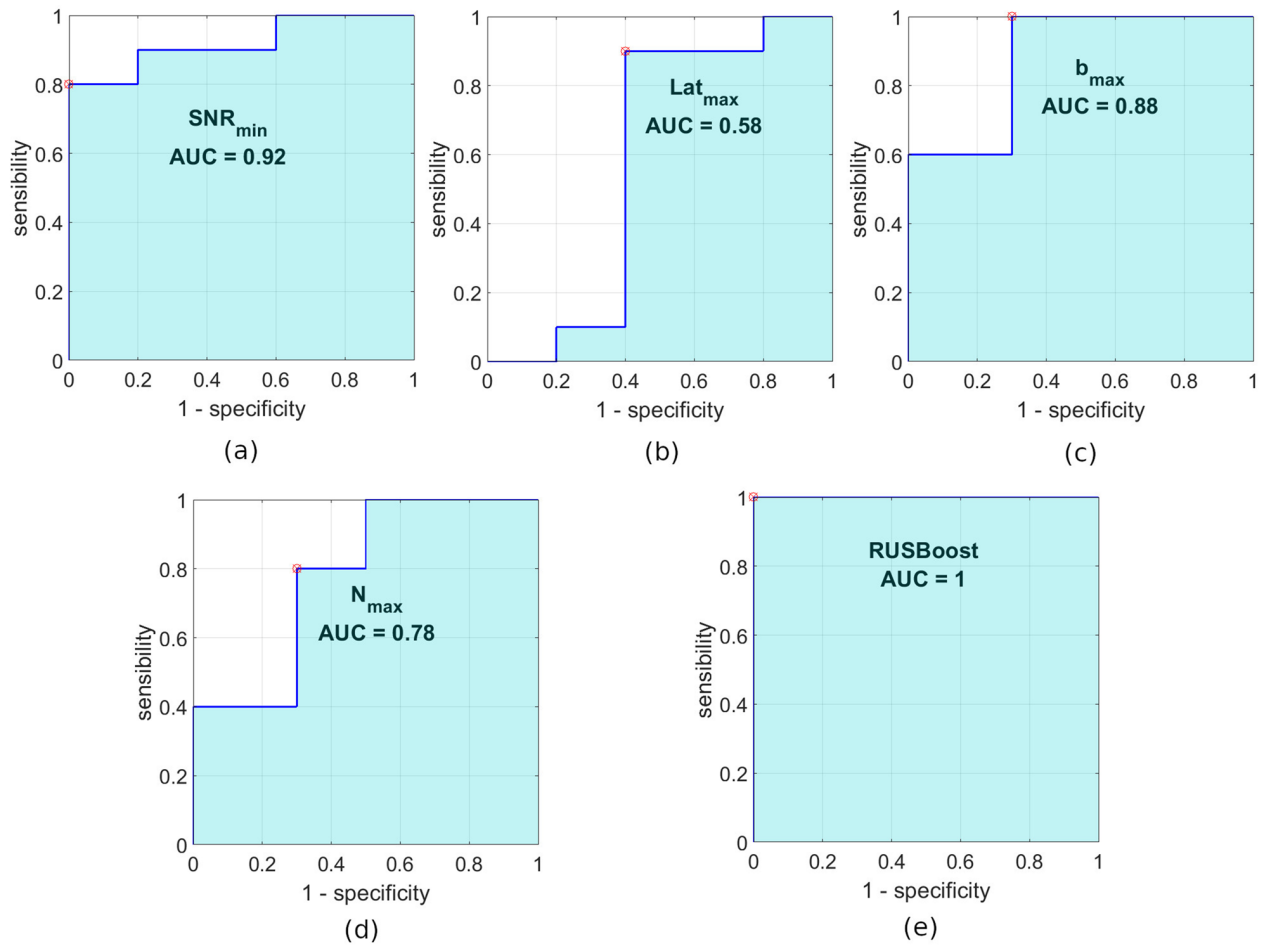
ROC graph of the variables and classifier. (**a**) ROC of SNR_min_. (**b**) ROC of Lat_max_. (**c**) ROC of b_max_. (**d**) ROC of N_max_. (**e**) ROC of the RUSBoosted Trees classifier. These images were generated using Matlab (Version: R2018b, Url: https://www.mathworks.com/products/matlab.html.

After testing all the possible combinations, the best results in the classifier were achieved by using as inputs the two variables with the greatest discriminant capacity: **SNR**_**min**_ and **b**_**max**_. The RUSBoosted Trees classifier was implemented in the Matlab Classification Learner Application (MathWorks, Natick, MA) with cross-validation folds = 15 folds. Cross-validation^26^ protects against overfitting by partitioning the dataset into folds and estimating accuracy on each fold. The results of classification using this system on our database are perfect, obtaining sensitivity = specificity = AUC_CLASSIFIER_ = 1. Figure 4 shows the ROC plot for the 4 variables analysed and for the classifier implemented.

## Discussion

Scientific work-up of patients with RIS may be a key step to expanding our knowledge of the pathogenesis of MS. This study provides a first insight into the afferent system of the visual pathway in RIS patients by analysing mfVEP responses and by describing and characterizing new parameters in the mfVEPs’ waveforms in order to monitor RIS progression.

Several studies have already shown that the mfVEP can be more sensitive in detecting optic nerve abnormalities than automated visual perimetry or OCT in both affected and unaffected eyes of CIS and MS subjects^27–29^. MfVEP latency and amplitude have been used as surrogate markers of demyelination and axonal loss in MS, respectively^30–33^. MfVEP amplitude has been shown to be a functional biomarker of axonal loss in MS^34^ while prolonged latencies in CIS subjects who have presented with optic neuritis (ON) are associated with increased risk of developing clinically definite MS^35^. In this regard, mfVEP response latency and amplitude show myelin and axonal integrity respectively. Hence, reduced amplitude reveals retinal ganglion cell or axonal loss whereas longer mfVEP latency indicates demyelination. Notwithstanding, latency, amplitude and structural retinal changes are all intimately related; thus, greater demyelination or prolonged latency may give on to more axonal degeneration due to loss of the metabolic support^28^.

The results of this study provide an insight into visual pathway neurodegeneration in RIS and new electrophysiological predictive factors to help monitor disease progression in RIS subjects. Analysis of the mfVEP recordings obtained in this study was performed in both the time domain (amplitude and latency) and the CWT domain (b_max_ and N_max_ variables). We observed that greatest diagnostic accuracy as regards progression was achieved with the SNR_min_ $\left( {{\text{AUC}}_{{{\text{SNR}}_{{{\text{min}}}} }} = 0.92} \right){ }$ variable, although in terms of the mean value the two CWT variables show greater capacity to identify those patients who will evolve clinically. This is one more example of how performing wavelet analysis on a bioelectric signal allows us to calculate new features that complement the classic biomarkers obtained in the time domain^19,36^. What is more, the best results in the automatic classifier (Fig. 4) are obtained by combining an input in the time domain (SNR_min_) with another in the CWT domain (b_max_).

Long-term prospective follow-up of patients with RIS is still rather limited. In our study, one third of our patient cohort progressed clinically, in line with the findings published in other papers. One of these first studies^12^ revealed that about 34% of RIS patients developed MS within 5 years; similarly, another study^37^ observed that 26.7% of their RIS patients converted to MS at 4.2 ± 1.4 years of follow-up. In a larger trial^7^, 128 out of 453 (28.2%) RIS-diagnosed subjects evolved to symptomatic MS. These differences in RIS progression between published studies could be explained by differences in the duration of follow-up, the population studied and the diagnostic tests used^38^.

The capacity to correctly identify and predict the evolution of those RIS patients at greatest risk of clinical progression is of great interest, as a significant cohort of them will progress over time to more advanced forms of demyelination. Younger age at RIS diagnosis, sex (male), higher number of MRI T2-hyperintense lesions, presence of spinal and gadolinium-enhanced MRI lesions and abnormal conventional VEPs have been associated previously with an increase in the risk of progression^7,8,39–41^.

Our study showed that the functional deficit in the visual afferent pathway detected by mfVEPs in our RIS cohort is in line with the structural loss in the retina observed in recent studies using OCT where retinal nerve fibre layer (RNFL) thickness and ganglion cell inner plexiform (GCIPL) layer thinning have been associated with clinical progression in RIS^2^.

Later generation spectral-domain OCTs have improved resolution and reliability to small RNFL changes, in addition to being able to assess the ganglion cell-inner plexiform (GCIPL) retinal layers, and also by utilizing the intereye difference of the GCIPL and RNFL. Likely, It’s expected new generation spectral-domain OCT could achieve multiple aims regarding diagnosis, prognosis, and treatment monitoring in RIS and related disorders^42^.

We acknowledge that our results should be externally validated in other larger cohorts and that combination with other biomarkers identified in the literature (based on MRI, cognitive deficit, etc.) would likely increase prognostic value. In summary, a significant incidence of subclinical optic nerve involvement was detected in RIS eyes by means of mfVEP and our results indicate that the use of advanced analysis of mfVEP signals may help identify those high-risk RIS subjects who will progress clinically to more advanced forms of demyelinating pathology.

## Data Availability

The datasets generated during and/or analysed during the current study are available from the corresponding author on reasonable request.
